# A comparative study on biosynthesized silver nanoparticles from *H. undatus* fruit peel and their therapeutic applications

**DOI:** 10.1186/s11671-024-03995-w

**Published:** 2024-03-18

**Authors:** Aswini Anguraj, Helan Soundra Rani Michael, Sathish Sugumaran, Gogul Ramnath Madhusudhanan, Rathish Kumar Sivaraman

**Affiliations:** 1 Department of Biotechnology, Sri Ramakrishna College of Arts and Science, Coimbatore, Tamilnadu 641 006 India; 2 Department of Physics, MVJ College of Engineering, Bengaluru, Karnataka India

**Keywords:** *H. undatus*, Antibacterial, Antifungal, Antioxidant, Thrombolytic, Anticancer activity

## Abstract

The green synthesis of nanoparticles (NPs) gained significant impacts in various fields due to the use of eco-friendly approaches. In this study, silver nanoparticles (AgNPs) were synthesized from the aqueous extract of *Hylocereus undatus* fruit peel. The presence of AgNPs was analysed using characterization methods such as UV‒Vis, FTIR, GCMS, XRD, EDAX, and FESEM. The synthesized AgNPs showed greater antibacterial activity against *Escherichia coli* than against *Streptococcus pneumoniae*. The antifungal activity against *Candida albicans* was greater than that against *Candida tropicalis.* The IC_50_ value for the antibiofilm activity of the AgNPs was 2.81 µg/mL, whereas that of the *H. undatus* peel extract was 1.34 µg/mL. The invitro antioxidant activity of the AgNPs was evaluated using two different methods. The DPPH radical scavenging activity of the AgNPs and fruit peel extract was observed with IC_50_ values of 3.8 and 2.03 µg/mL respectively. On the other hand, nitric oxide radical scavenging activities were recorded and the IC_50_ values were calculated to be 2.8 and 2.3 µg/mL. The AgNPs demonstrated thrombolytic activity in human blood with 10, 32.36, and 56.25% lysis. The cytotoxicity of the AgNPs was minimal, with an IC_50_ of 0.2 µg/mL and the peel extract had the greatest cytotoxicity with an IC_50_ of 0.3 µg/mL. The findings of this study demonstrated that the synthesized AgNPs from *H. undatus* peel extract could be potential candidates for treating prostate cancer.

**Graphical abstract:**

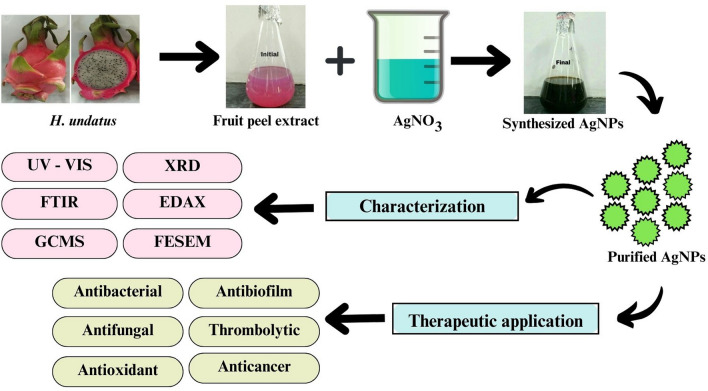

## Introduction

Nanotechnology is an emerging field that aims to create molecular, atomic and supramolecular properties [1]. It has gained importance in cosmetics, drug delivery, biomedical applications, cosmetics and cancer therapy [2]. Metallic NPs such as silver, copper oxide, zinc oxide, and gold NPs are synthesized extensively in nanostructured form [3]. Among the above-mentioned metals, AgNPs have attracted an increased amount of attention due to their unique characteristics. It can be synthesized by physical, chemical, and biological methods [4]. Biological entities such as bacteria, fungi, yeasts, and plants have been utilized in the synthesis of AgNPs, which are less toxic, less expensive, more stable, and more eco-friendly alternatives to chemical methods [5]. Among the available biological methods, microbe-mediated synthesis of NPs nanoparticles is not commercially feasible due to the high aseptic requirement. Plant-mediated AgNPs offer several advantages over microorganisms. Because these methods can be easily scaled up, they are more practical for large-scale production. The NPs can be effectively capped and reduced by extracting active components from plants such as flavonoids, alkaloids, terpenes, tannins, and saponins [6]. AgNPs from plant extracts have a wide range of applications in the fields of food, health, agriculture, disease control, pharmaceuticals, and medicine [7]. AgNPs have shown potential antibacterial, antifungal, anti-inflammatory, antioxidant, antiviral, anticancer, antiplasmodial, and anti-platelet effects [8].

Prostate carcinoma (PC) is the primary cause of cancer in the male reproductive system and affects a significant number of newly diagnosed cancer patients, accounting for approximately 25% of cases. When combined with colorectal cancer, PC has become the second leading cause of cancer-related deaths in males in the US, following lung and bronchus cancer. Approximately 80% of prostate cancer cases are found in the peripheral zone, while 10–20% of prostate cancer cases occur in the transition zone [9]. Treatment methods such as cholesterol-lowering drugs, biopsies, and conventional heating techniques can be costly and may have side effects such as pain or discomfort due to unwanted reflection, scattering, or absorption [10]. It is crucial to explore alternative treatments or medications to address these limitations. NPs have shown promise in the treatment of prostate cancer patients [11]. The toxicity associated with this treatment is moderate and only temporarily affects quality of life. Hence, there is a need to discover new cancer therapies that are both biocompatible and cost-effective [12].

Dragon fruit (*Hylocereus undatus*) is a cactus species that is commonly consumed in Asian countries. It is a tropical fruit with a red peel and green pins. It is a great source of low-calorie dietary fibre [13]. It contains antioxidants that can help to prevent various diseases such as cancer, cardiovascular issues, diabetes, and urinary and gastrointestinal problems [14]. The peel of dragon fruit is composed of pectin, phyllocactin, steroids, betacynanin, and betanin. Previous studies have reported that it has high antioxidant, antimicrobial, cardioprotective, anti-inflammatory, and anticancer properties [15]. In this study, we focused on the use of *H. undatus* for the bio-reduction of silver ions in an aqueous medium which leads to the formation of silver nanoparticles. The synthesized nanoparticles were characterized and evaluated for antibacterial, antifungal, antioxidant, and thrombolytic activity. Additionally, we examined the potential of these AgNPs to inhibit the growth of prostate cancer cells.

## Materials and methods

### Sample collection and extraction

In the present study, *H. undatus* was collected from the local market, R. S. Puram, Coimbatore, India. The peel of *H. undatus* was washed with double distilled water until all the impurities were removed and kept after which the sample was dried in a sterile environment. The dried samples were finely ground into powder and further processed for extraction.

### Preparation of aqueous extract

The *H. undatus* aqueous extract was prepared by boiling 20 g of powdered peel in 500 mL of distilled water for 15 min. The extracts were agitated and covered until they reached room temperature. The crude extract was filtered through Whatman filter paper No.1. The resulting extract was stored in a container at 2 °C for further study.

### Synthesis of AgNPs

A reaction mixture consisting of 220 mL of dragon fruit peel extract and 110 mL of 10 mM silver nitrate was stirred for 24 h at room temperature using a magnetic stirrer. The reaction mixture was then centrifuged at 12,000 rpm for 15 min at 4 °C after which the supernatant was discarded. The resulting pellet was dissolved in distilled water and centrifuged again under the same conditions as mentioned before to ensure the purity of the nano-silver. The resultant pellet was dried and stored for further characterization [16].

### Characterization of silver nanoparticles

The synthesized AgNPs were characterized using a UV–visible spectrophotometer, Fourier Transform Infrared Spectrophotometer (FTIR), Gas Chromatography-Mass Spectrometry (GCMS), X-ray Diffraction (XRD), Energy Dispersive X-ray Spectroscopy (EDAX), and Field Emission Scanning Electron Microscopy (FESEM).

### Antibacterial activity

The disc diffusion method was used to evaluate the antibacterial activity of AgNPs against both Gram-positive (*Streptococcus pneumoniae*) and Gram-negative (*Escherichia coli*) bacteria. The bacterial strains were spread on nutrient agar plates using a sterile cotton swab. The disc was treated with two distinct concentrations (40 and 80 µL) of synthesized AgNPs. Kanamycin was used as a positive control and distilled water was used as a negative control. Then the plates were incubated at 37 °C for 24 h. The antibacterial activity was evaluated by measuring the zone of inhibition [17]. The experiments were replicated three times and the diameter of the zone of inhibition was reported as the mean ± standard deviation.

### Antifungal activity

The antifungal potential of the biosynthesized AgNPs against two pathogenic strains *Candida albicans* and *Candida tropicalis* was evaluated using the agar well diffusion method as described by Joseph et al. [18]. Wells with a diameter of 8 mm were punched in potato dextrose agar medium and 100 µL of each microorganism was spread using a sterile cotton swab. Distilled water was used as the negative control while Voriconazole was used as the positive control. Two concentrations 40 and 80 µL of AgNPs were added to the wells and incubated at 37 °C for 24 h. The zones were subsequently determined and recorded.

### Antibiofilm activity

A 96-well microtiter plate was used to evaluate the antibiofilm effectiveness of the AgNPs and *H. undatus* peel extract. The wells of the plates were filled with 180 μL of Muller Hinton broth and 10 μL of the test pathogens. Then 10 μL of AgNPs and *H. undatus* peel extract were added and the mixture was thoroughly mixed. The test plates were then incubated for 24 h at 37 °C. After incubation, the microtiter plates were washed with phosphate-buffered saline (PBS) to remove non-adherent bacteria. The plates were air-dried for 45 min, after which the protein wells were fixed with sodium acetate. Crystal violet stain was added and the samples were incubated in the dark for 30 min. Excess dye was removed by washing with water. The plates were air-dried, 200 μL of ethanol was added to each well and absorbance was measured at 620 nm [19]

### Thrombolytic activity

The thrombolytic activity of the AgNPs and the peel extract of *H. undatus* was evaluated according to the method of Devi et al. [20] with slight modifications. After the weight of the empty Eppendorf tube was determined 20, 40, and 60 μL of blood were drawn in different tubes and incubated at 37 °C for 30 min. After clot formation, the tubes were reweighed to determine the weight of the blood clot (X). This was achieved by subtracting the initial weight of the tube. 100 µL of AgNPs at two different concentrations (500 and 1000 µg/mL) was added to the tubes and incubated at 37 °C for 90 min. To calculate the weight of the non-lyzed blood clot (Y), the initial weight of the tube was subtracted from the final weight of the tube, which contained the remaining blood clot. 100 µL of distilled water was used as a negative control and streptokinase was used as a positive control. Thrombolytic activity was calculated using the following equation

$${\text{Thrombolysis }}\left( \% \right) \, = {\text{ X }}{-}{\text{ Y}} \times {1}00/{\text{X}}$$

### Antioxidant activity

#### DPPH radical scavenging assay

The scavenging ability of the AgNPs and *H. undatus* peel extract was determined using the DPPH method described by Phull et al. [21]. The DPPH assay was performed in a 96-well plate having ascorbic acid as the reference standard. AgNPs and *H. undatus* peel extracts (20, 40, 60, 80, and 100 μL) were removed from the stock solution and added to each well of the plate. Then, 100 μL of the DPPH solution was added and incubated at 27 °C for 20 min. The absorbance of the reaction mixture was measured at 517 nm using a microplate reader. The scavenging activity was calculated as

$$\% {\text{ DPPH}}\;{\text{radical}}\;{\text{scavenging}}\;{\text{activity}} = {\text{control}}\;{\text{OD}} - {\text{sample}}\;{\text{OD}}/{\text{control}}\;{\text{OD}} \times {1}00$$

#### Nitric oxide radical scavenging activity

The Nitric oxide radical scavenging activity of the AgNPs and *H. undatus* peel extract was determined using the method outlined by Makhija et al. [22] with slight modifications. Various concentrations of the AgNPs and *H. undatus* peel extract (20, 40, 60 80, and 100 μL) were combined with 3 mL of sodium nitroprusside (10 mMol/L) in phosphate buffered saline (0.2 mMol/L, pH 7.4). The mixture was then incubated at 25 °C for 150 min. Then, 500 μL of Griess reagent (composed of 2% orthophosphoric acid, 1% sulphanilamide, and 0.1% N-1-naphthyl ethylenediamine dihydrochloride) was added. The absorbance was measured at 546 nm. All measurements were conducted in triplicate and the mean values were calculated.

$${\text{Nitric}}\;{\text{oxide}}\;{\text{scavenged }}\left( \% \right) \, = {\text{ A}}_{{{\text{control}}}} {-}{\text{ A}}_{{{\text{test}}}} /{\text{ A}}_{{{\text{control}}}} \times { 1}00$$

### Cytotoxicity assay

#### Cell culture

The human prostate cancer cell line (PC3) was purchased from NCCS, Pune. The cells were cultured in Dulbecco’s Modified Eagle Medium (DMEM) supplemented with 10% Fetal Bovine Serum (FBS) and 1% antibiotics. The cells were kept at a temperature of 37 °C in 5% CO_2_ and 95% relative humidity [23].

#### MTT assay

The cytotoxic effects of the AgNPs and *H. undatus* peel extract were evaluated via an MTT assay. PC3 cells were seeded in a 96-well plate and incubated for 24 h, then 200 µL of DMEM supplemented with 10% FBS. Doxorubicin an anticancer drug was used as standard, whereas the untreated cells were used as a negative control. Various concentrations (12, 25, and 55 µg/mL) of AgNPs and *H. undatus* peel extract were added and incubated for 48 h. After the addition of MTT, the cells were again incubated at 37ºC for 4 h. The media was then removed, 200 µL of DMSO was added to each well and the absorbance was read at 570 nm [24].

### Statistical analysis

The descriptive statistics were performed for the in vitro assays based on the mean average method and the standard errors were calculated.

## Results and discussion

### Characterization of AgNPs

**Fig. 1:**
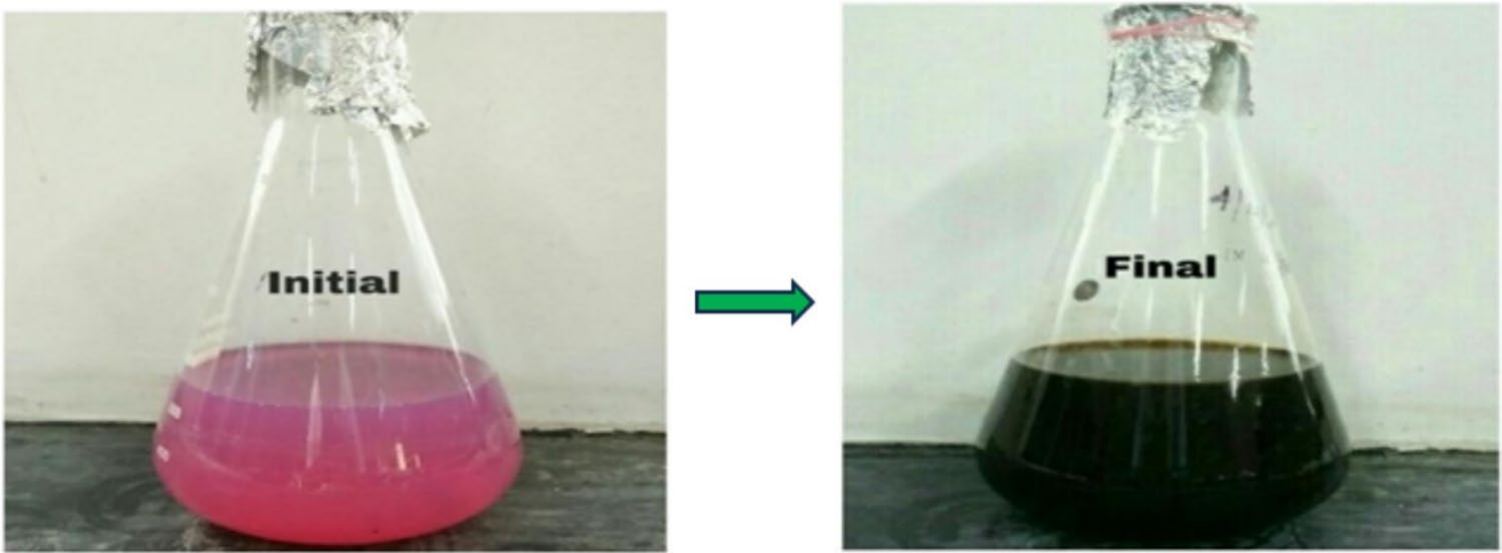
The colour change of the solution after the reduction of AgNO_3_ by *H. undatus* fruit peel extract

**Fig. 2:**
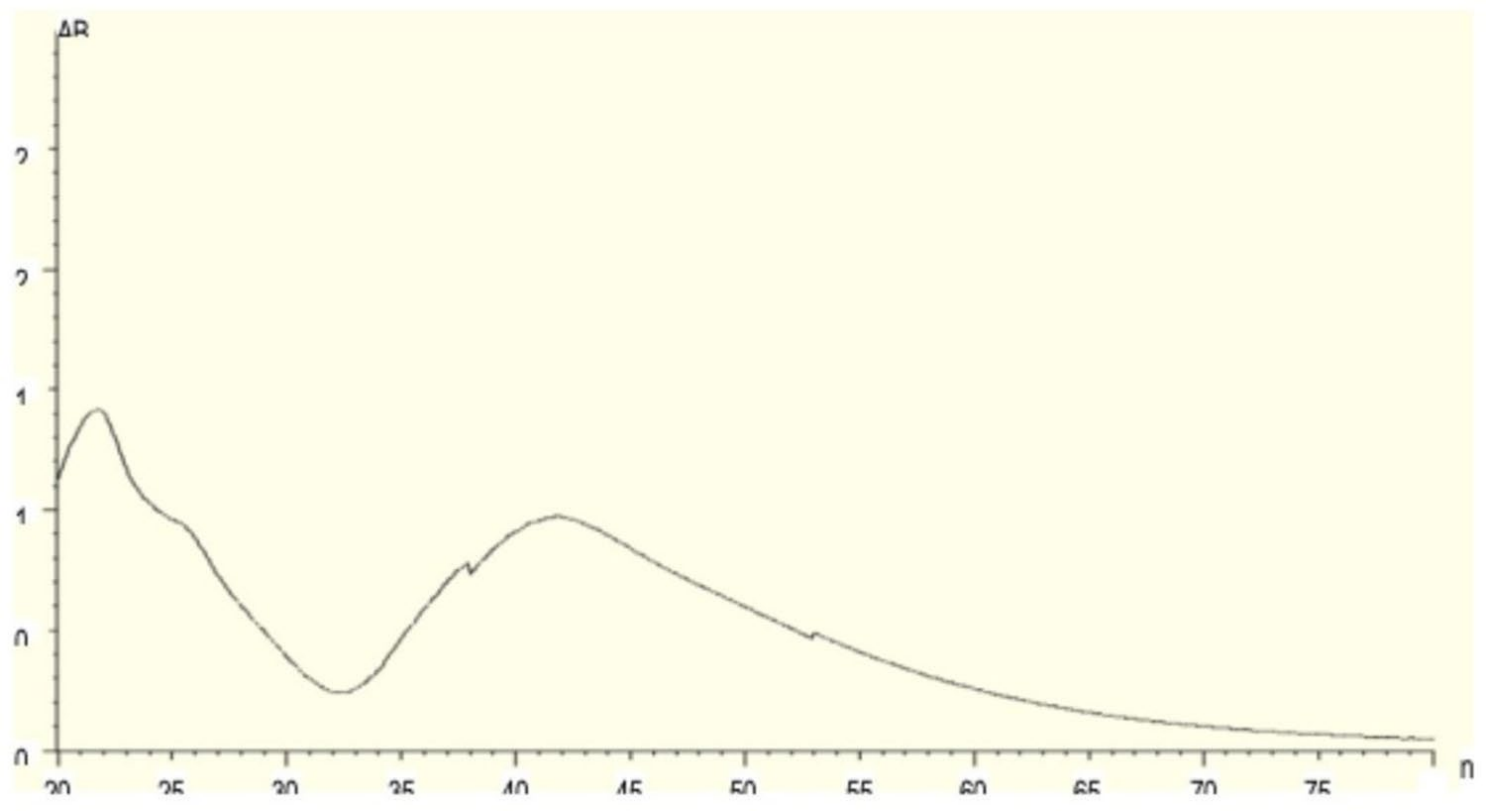
UV–Visible spectra of AgNPs biosynthesized from *H. undatus* fruit peel extract

The present study revealed that extract of *H. undatus* fruit peel can be used to convert the silver in the form of nitrate. This interaction resulted in the formation of a pale pink to dark brown indicating the presence of AgNPs [25] as shown in Fig. 1. Similar color changes were also observed in a previous study [16]. The UV–Vis spectra were recorded after time intervals of 20 min, 25 min, 30 min, 35 min, 49 min, 45 min, 50 min, 55 min, 60 min, 65 min and 70 min. Figure 2 shows the absorption spectra of the AgNPs obtained from *H. undatus* fruit peel extract. The UV spectrum exhibited a strong peak at 417 nm which confirmed the formation of AgNPs.

**Fig. 3:**
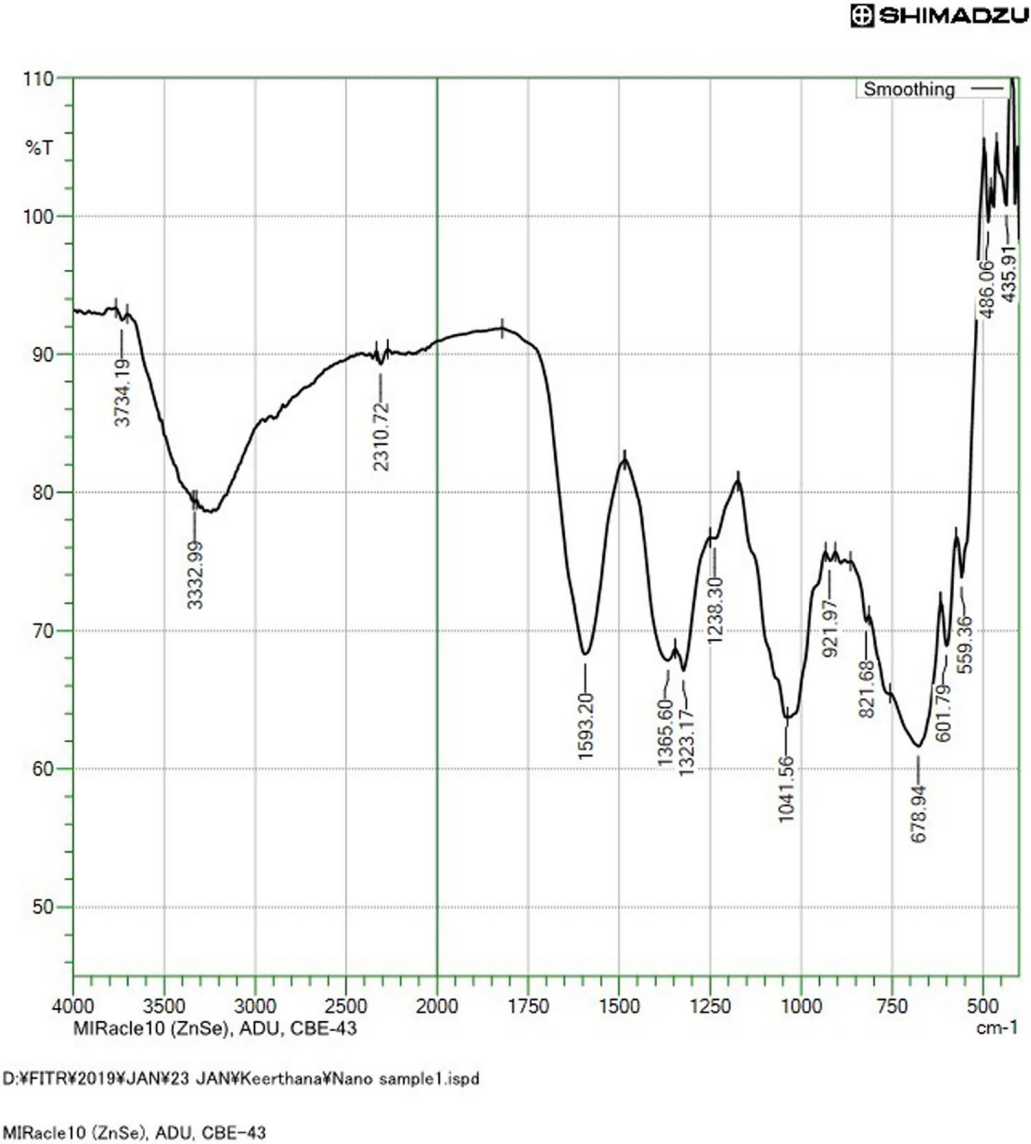
FTIR analysis of the biosynthesized AgNPs using *H. undatus* fruit peel extract

The purity of the synthesized NPs was checked by FTIR analysis within the range of 400–4000 cm^−1^ [26]. Figure 3 shows the FTIR spectra of the biosynthesized AgNPs and the absorption peak bands at 3734.19, 3332.99, 2310.72, 1593.20, 1365.60, 1323.17, 1238.30, 1041.56, 921.97, 821.68, 678.94, 601.79 and 559. 36 cm^−1^. These absorbance bands are known to be associated with the stretching vibrations of O–H (alcohols), O–H (alcohols), C=N (nitriles), C–C (aromatics), C–H (alkanes), C–N (aromatic amines), C–N (aliphatic amines), C–O (alcohols), O–H (carboxylic acids), C–Cl (alkyl halides), C–Br (alkyl halides), C–Br (alkyl halides), C–Br (alkyl halides). *H. undatus* fruit peel is primarily composed of betanin, pectin, hylocerenin, betacyanin, and phyllocactin [27]. This study confirmed the presence of a significant amount of phytocompounds and these substances may be responsible for the reduction, capping, and synthesis of AgNPs.

**Fig. 4:**
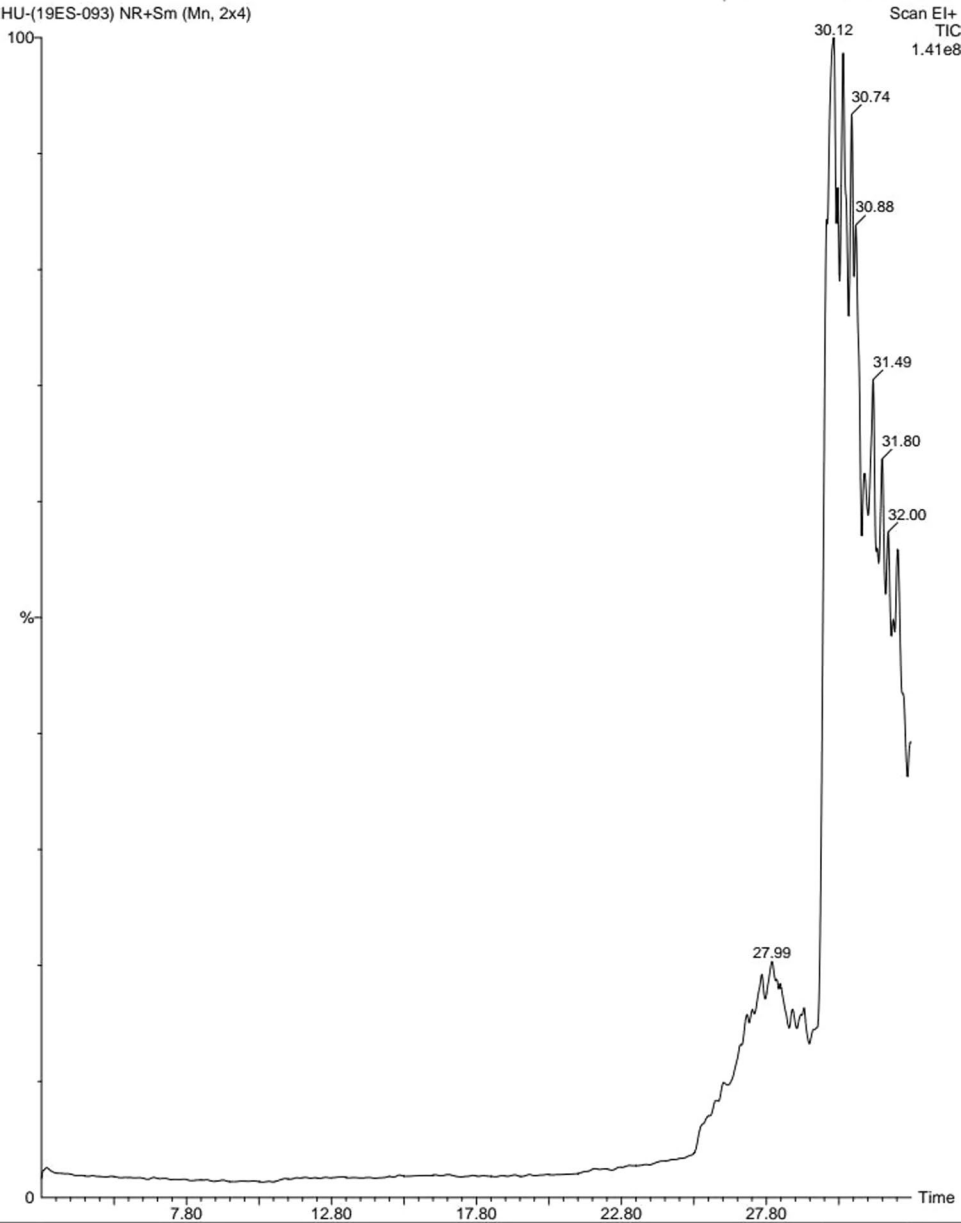
GC–MS analysis of AgNPs biosynthesized from *H. undatus* fruit peel extract

GC–MS analysis of the peel extract of *H. undatus* was also conducted to determine the bioactive chemical compounds present in the extract. Figure 4 displays the GC–MS spectrum with peaks and retention times. The peel extract of *H. undatus* contains seven major bioactive compounds namely 6-methyl-2, (4-bromophenyl)-7-phenylmethylindolizine, N-[(4,6-Dimethoxynaphthalen-1-yl) methylene], 2,5-dichloro-4-hydroxyphenylamine, oxacycloheptadec-8-en-2-one, androstan-17one, 3-ethyl-3-hydroxy, pyridinium-3-carboxamide, 6-chloro-4-trifluoromethyl-N-[2,4-dichloeo-6-methyl]-N-methyl, 1,2-Dipalmitoyl 3-acetyl glycerol gallic acid, and triamcinolone acetonide. The peak intensity of N-[(4,6Dimethoxynaphthalen-1-ylmethylene] and 2,5-dichloro-4-hydroxyphenylamine were greater with a retention time of 30.12 min followed by Oxacycloheptadec-8-en-2-one with a retention time of 30.74 (Table 1).

**Table 1:** Chemical composition of the *H. undatus* peel extract

S. no.	RT	Compound name	Molecular formula	Molecular weight	Peak area %
1	27.99	6-methyl-2, (4-bromophenyl)-7-phenylmethylindolizine	C_22_H_18_BrN	376.289	7.991989
2	30.12	N-[(4,6Dimethoxynaphthalen-1-ylmethylene], 2,5-dichloro-4- hydroxyphenylamine	C_19_H_15_Cl_2_NO_3_	376.233	8.799596
3	30.74	Oxacycloheptadec-8-en-2-one	C_16_H_28_O_2_	252.392	15.430418
4	30.88	Androstan-17one 3-ethyl-3-Hydroxy	C_21_H_34_O_2_	318.49346	13.414466
5	31.49	Pyridine-3-carboxamide, 6- chloro-4-trifluoromethyl-N-[2,4-dichloeo-6-methyl]-N-methyl	C_15_H_10_C_13_F_3_N_2_O	397.606	11.854260
6	31.80	1,2-Dipalmitoyl 3-acetylglycerol Gallic acid	C_37_H_70_O_6_	610.948	11.854137
7	32.00	Triamcinolone acetonide	C_24_H_31_FO_6_	434.497	10.116891

**Fig. 5:**
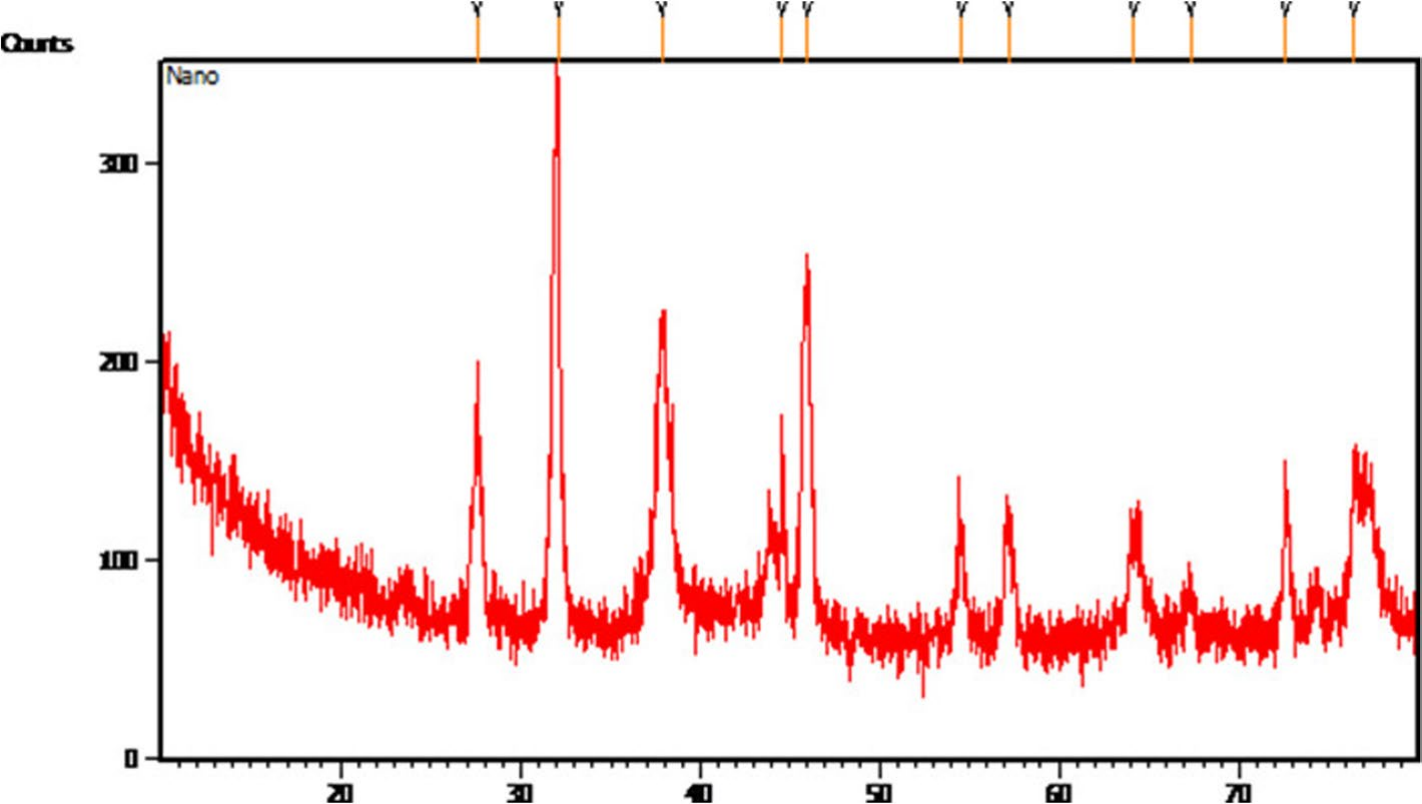
XRD analysis of AgNPs biosynthesized from *H. undatus* fruit peel extract

The synthesized AgNPs were characterized using XRD to confirm the presence of silver ions and to determine the structure [28]. Figure 5 shows the XRD pattern of the AgNPs, which confirms the crystalline nature of synthesized the AgNPs. The 2θ peaks at 27.57, 32.09, 37.88, 44.65, 46.02, 54.59, 57.19, 64.16, 67.32, 72.62, and 76.42 cm^−1^ correspond to the crystalline planes of the face-centered cubic structure of metallic silver. The intense peak at 32.09 cm^−1^ possibly suggested the presence of silver ions as the major constituent in the biosynthesized AgNPs. A similar result was observed by Phongtongpasuk et al. [16] who identified the most intense peak at 32.5 cm^−1^ indicating the presence of AgNPs.

**Fig. 6:**
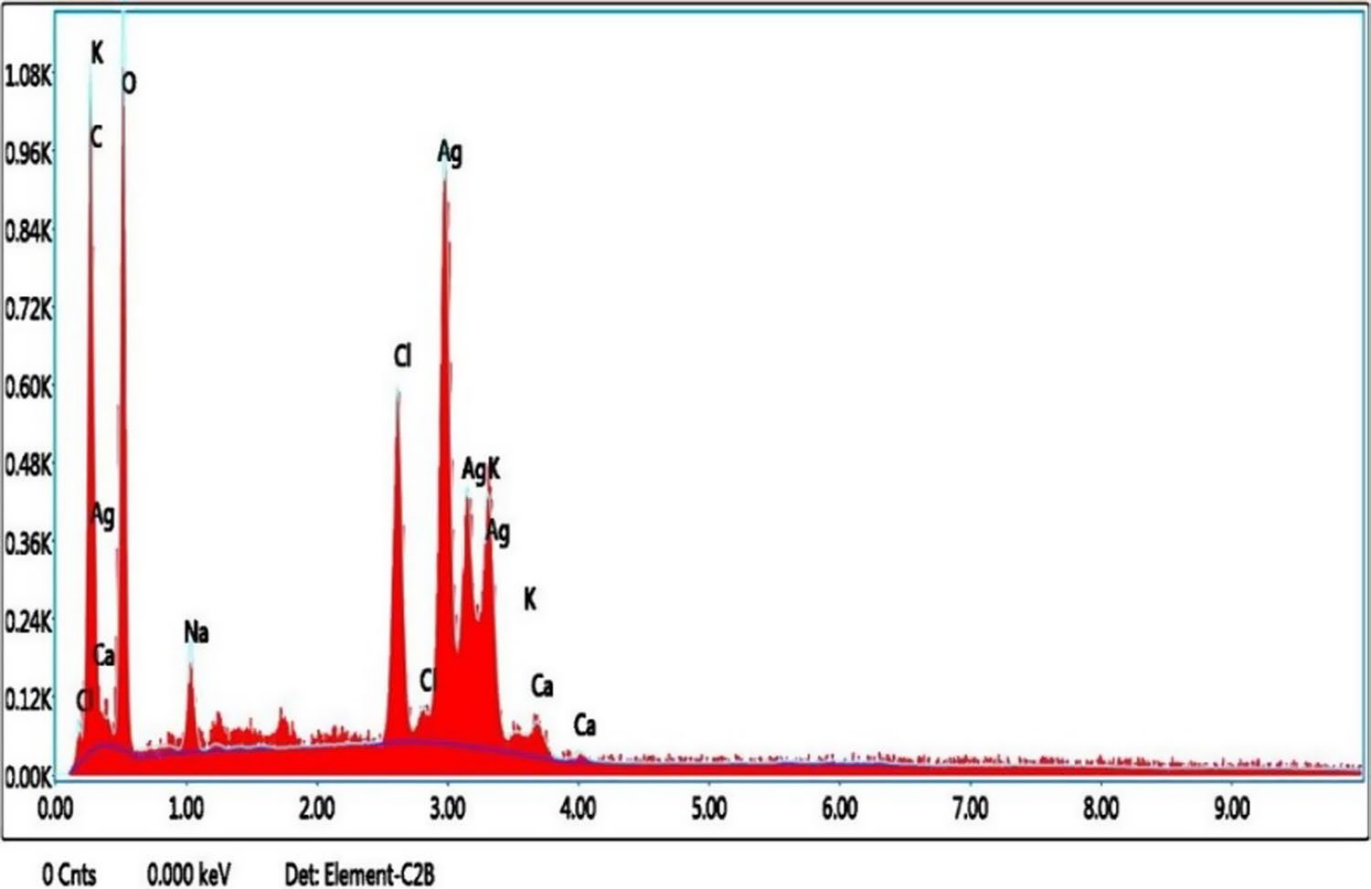
EDAX analysis of AgNPs biosynthesized from *H. undatus* fruit peel extract

The elemental analysis of the material is depicted in Fig. 6, which shows the synthesized AgNPs through the EDAX spectrum [29]. This analysis demonstrated a prominent indication of a metallic silver area at 3 keV and confirmed the formation of silver nanoparticles synthesized through the utilization of *H. undatus* peel (Fig. 6). The Ag peak showed a weight percentage of 35.12 and an atomic percentage of 8.02. A low calcium signal was observed and five moderate signals for carbon, oxygen, sodium, chloride, and potassium were detected as a result of the chemicals used in the sample preparation.

**Fig. 7:**
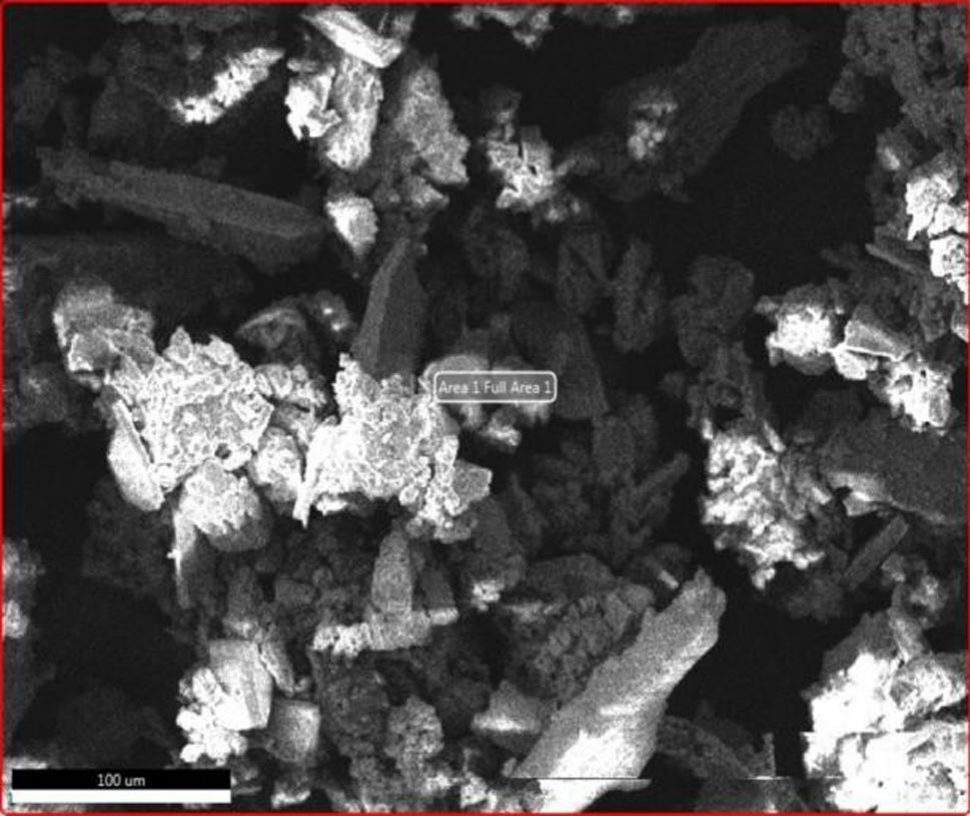
FESEM analysis of AgNPs biosynthesized from *H. undatus* fruit peel extract

FE-SEM images of AgNPs synthesized from the fruit peel extract of *H. undatus* are shown in Fig. 7. The surface morphology of the AgNPs was spherical with agglomeration. In the present study, the histogram of the particle size ranged from 52 to 79 nm. An increased concentration of bioactive compounds in colloidal solution could lead to the formation of nanoclusters. The signals produced from the interaction between the electrons and the sample provide valuable information regarding the sample's external morphology, chemical composition, crystalline structure, and material orientation.

### Antibacterial activity

**Fig. 8:**
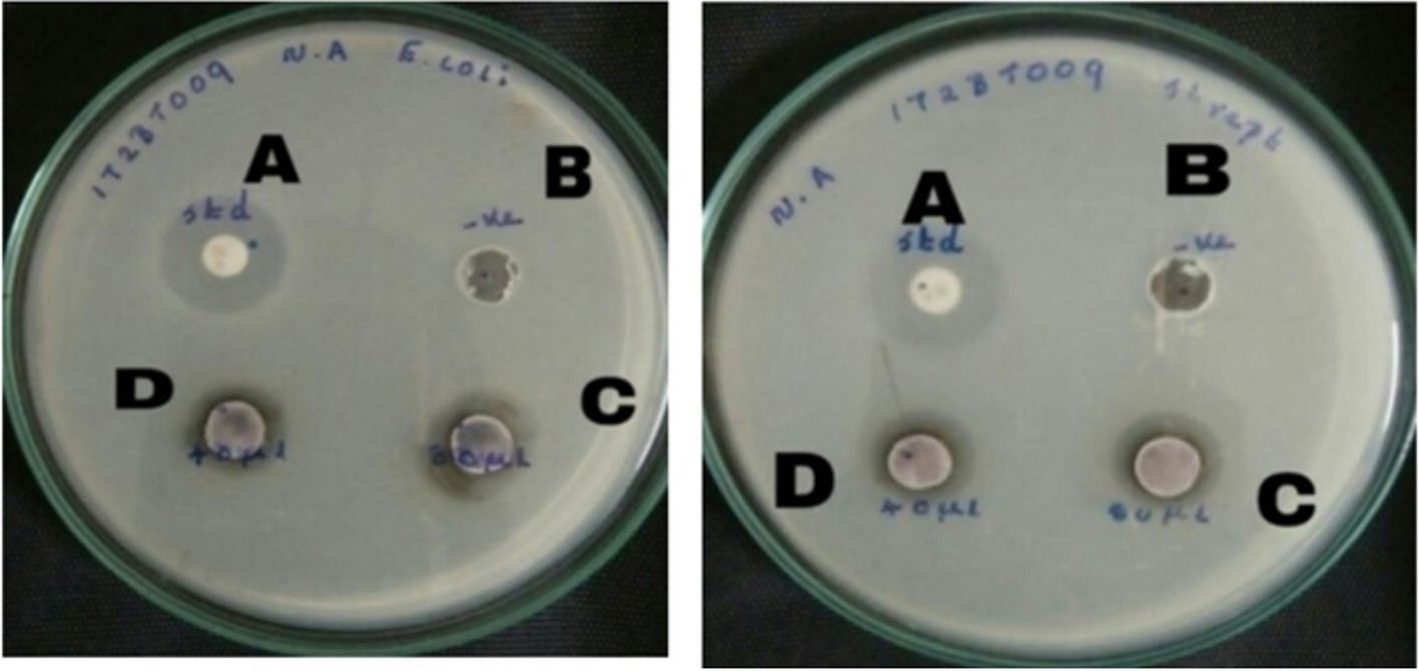
Biosynthesized AgNPs showing a clear zone of inhibition against *E. coli* and *S. pneumoniae*, **A** Positive control—Kanamycin, **B** Negative control—Distilled water, **C** 80 µL of AgNPs treated, **D** 40 µL of AgNPs treated

The antibacterial activity of the synthesized AgNPs was tested at various concentrations by the disc diffusion method. Figure 8 illustrates the zone of inhibition around the individual bacterial culture. The zones of inhibition were measured to be 1.2 ± 0.2 and 1.6 ± 0.1 cm for *E. coli* while in the case of *S. pneumoniae* 1.1 ± 0.1 and 1.3 ± 0.1 cm were measured at concentrations of 40 µL and 80 µL respectively, of synthesized the AgNPs. In the present study, the synthesized AgNPs showed greater antibacterial activity against *E. coli* than against *S*. *pneumoniae*. The potential cause of this occurrence could be attributed to the presence of phytochemicals within *H. undatus.* The enhanced antibacterial efficacy of AgNPs can be predominantly attributed to the synergistic interplay between the aforementioned nanoparticles and the naturally occurring chemicals found within the extract as previously substantiated in a prior investigation. It has been determined that AgNPs can liberate silver ions within bacterial cells, thus enhancing their bactericidal properties [30].

### Antifungal activity

**Fig. 9:**
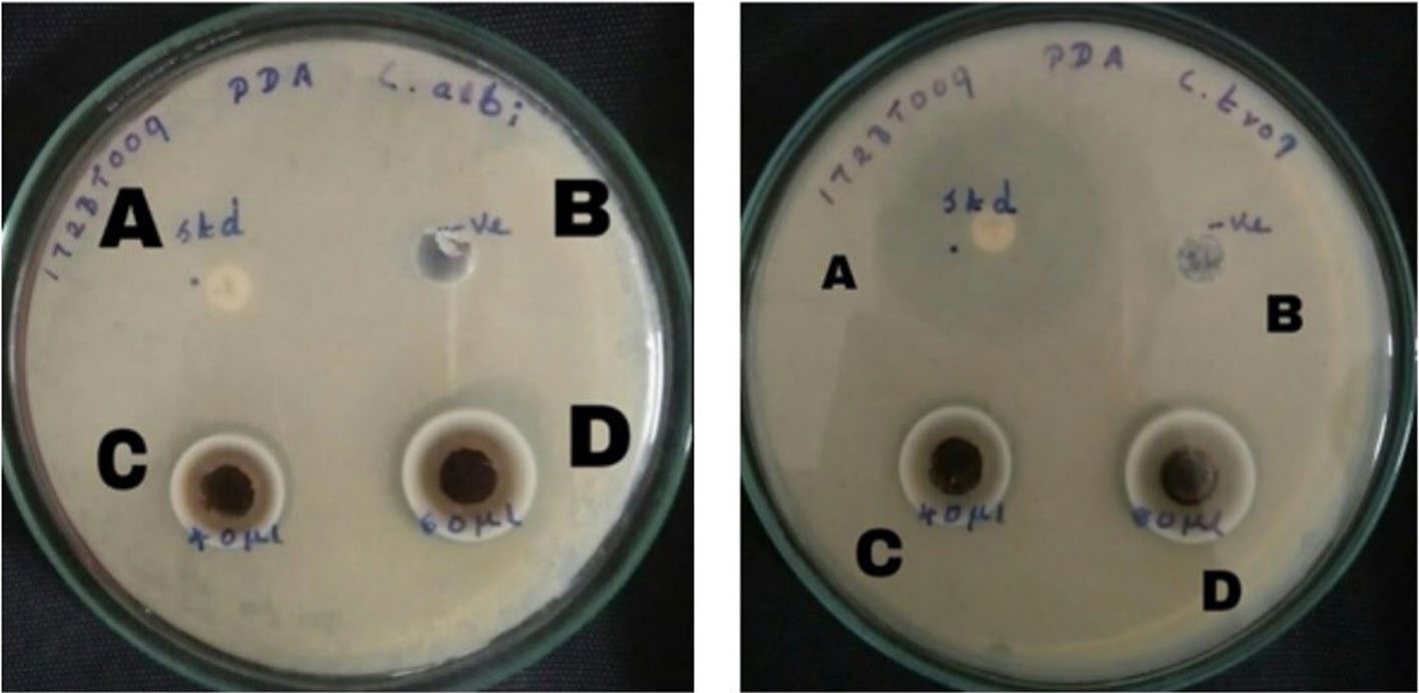
Antifungal activity of AgNPs against *C. albicans* and *C. tropicalis*, **A** Positive control—Voriconazole, **B** Negative control—Distilled water, **C** 40 µL of AgNPs treated, **D** 80 µL of AgNPs treated

The antifungal potential of the biosynthesized AgNPs toward two pathogenic strains *Candida albicans* and *Candida tropicalis* was evaluated using the well diffusion method. The antifungal activity can be identified by the zone of inhibition (Fig. 9). The results indicate that the biosynthesized AgNPs possess significant antifungal activity against the two fungal strains that were tested. No zone of inhibition was observed in the control. The synthesized AgNPs that inhibited the fungal growth of *C. albicans* were found to be 1.1 ± 0.1 and 1.3 ± 0.3 cm in length, while those of *C. tropicalis* were measured to be 0.8 ± 0.2 and 1.2 ± 0.2 cm at a concentration of 40 and 80 µL respectively. The antifungal activity against *C. albicans* was greater than that against *C. tropicalis.* Numerous studies have shown the antifungal activity of biosynthesized AgNPs against filamentous fungi [31]. NPs can disrupt the cell walls and cell membranes of fungi, resulting in the release of intracellular components that may lead to fungal death [32]. Fungal death is also promoted by the production of hydroxyl radicals and reactive oxygen species [33].

### Antibiofilm activity

**Fig. 10:**
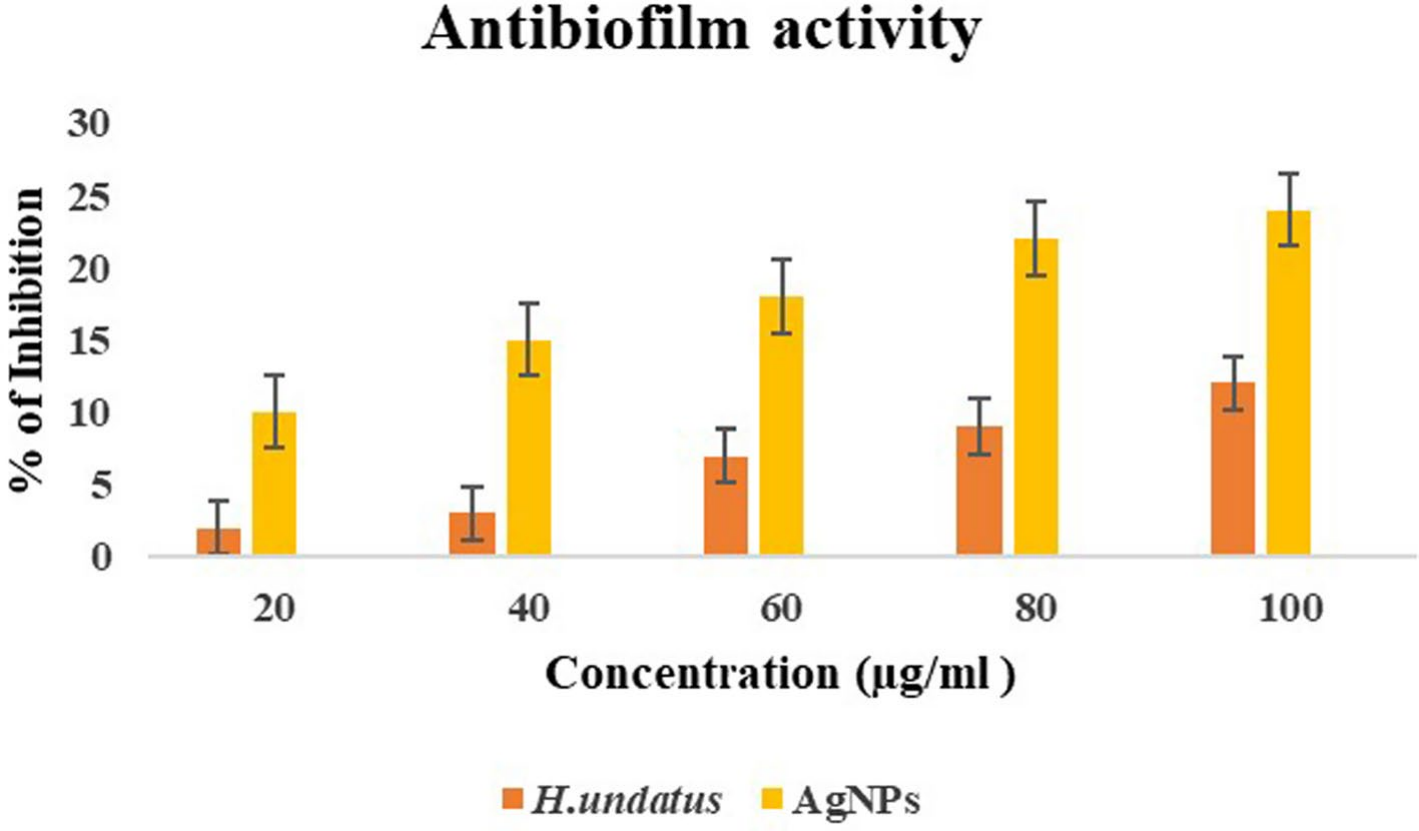
Determination of the % antibiofilm activity of AgNPs and *H. undatus* peel extracts on *E. coli*

According to the findings of the National Institutes of Health and Centre for Disease Control, approximately 65–80% of infections are caused by microbes that form biofilms. Among these, the most prevalent are gram-negative bacteria (*P. aeruginosa* and *E. coli*) and gram-positive bacteria (*Staphylococci* and *S. aureus*). AgNPs possess the unique capability to disrupt the biofilm formation of various pathogenic bacteria [29]. In this study, the ability of biosynthesized AgNPs and *H. undatus* peel extracts to inhibit biofilm formation in *E. coli* was examined. An ELISA was used to measure the absorbance at 620 nm and the obtained values were used as an indicator of bacterial adhesion to the cell wall surface for the formation of biofilms. The biofilm activities of the AgNPs and *H. undatus* peel extracts were compared based on their resulting IC_50_ values. The synthesized AgNPs exhibited maximum inhibitory activity against biofilms (IC_50_ 2.81 µg/mL) while the *H. undatus* peel extract exhibited an IC_50_ value of 1.34 µg/mL (Fig. 10).

### Thrombolytic activity

**Fig. 11:**
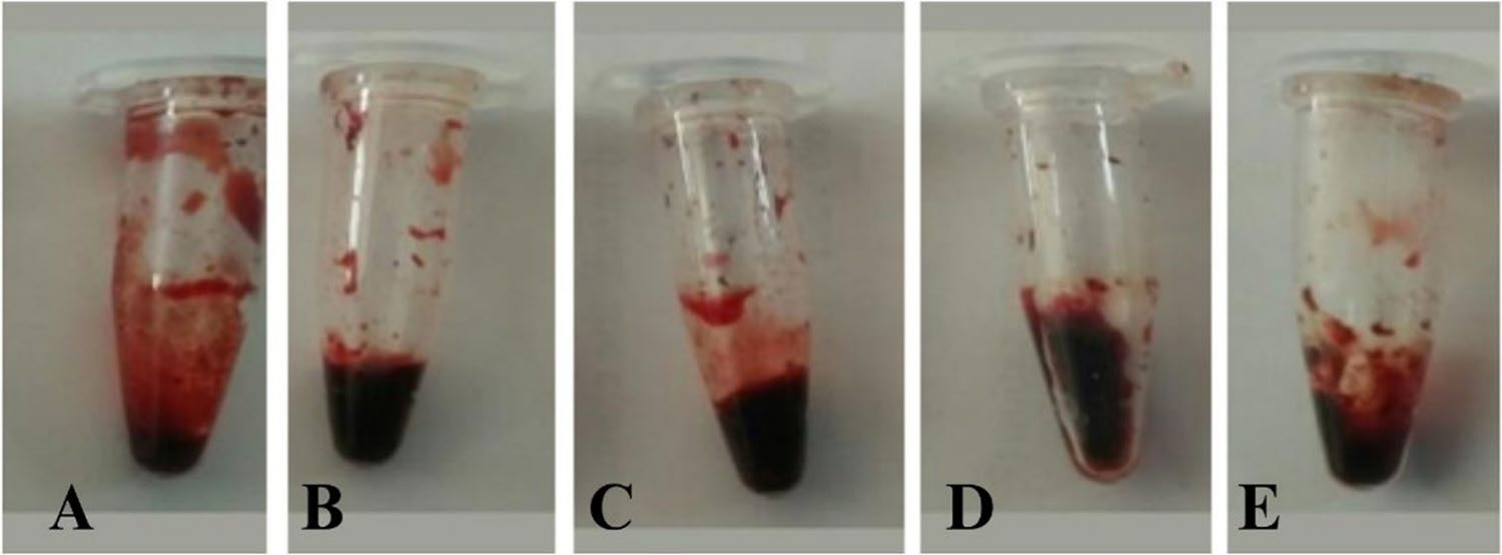
Thrombolytic activity of AgNPs and *H. undatus* peel extracts, **A** Positive control—Streptokinase, **B** Negative control—Distilled water, **C** 20 µg/mL of AgNPs, **D** 40 µg/mL of AgNPs, **E** 80 µg/mL of AgNPs

The thrombolytic activity of the AgNPs and *H. undatus* peel extracts was evaluated at three different concentrations. The percentages of thrombolysis were 10, 32.36 and 56.25% for the AgNPs solutions at the concentration of 20, 40, and 80 µg/mL respectively (Fig. 11). Distilled water was used as a negative control resulting in clot lysis of 6.07% while streptokinase was taken as a positive control exhibiting clot lysis of 50%. AgNPs may be involved in activating the enzymes that generate plasmin, an enzyme capable of breaking down the cross-links between fibrin molecules and dissolving blood clots [34]. Limited research has been conducted on the use of AgNPs as a thrombolytic agent. The study revealed that as the concentration of AgNPs increased and the percentage of clot lysis also increased the potential of AgNPs could be valuable applications in the clinical field for preventing thrombosis and other related disorders.

### Antioxidant activity

**Fig. 12:**
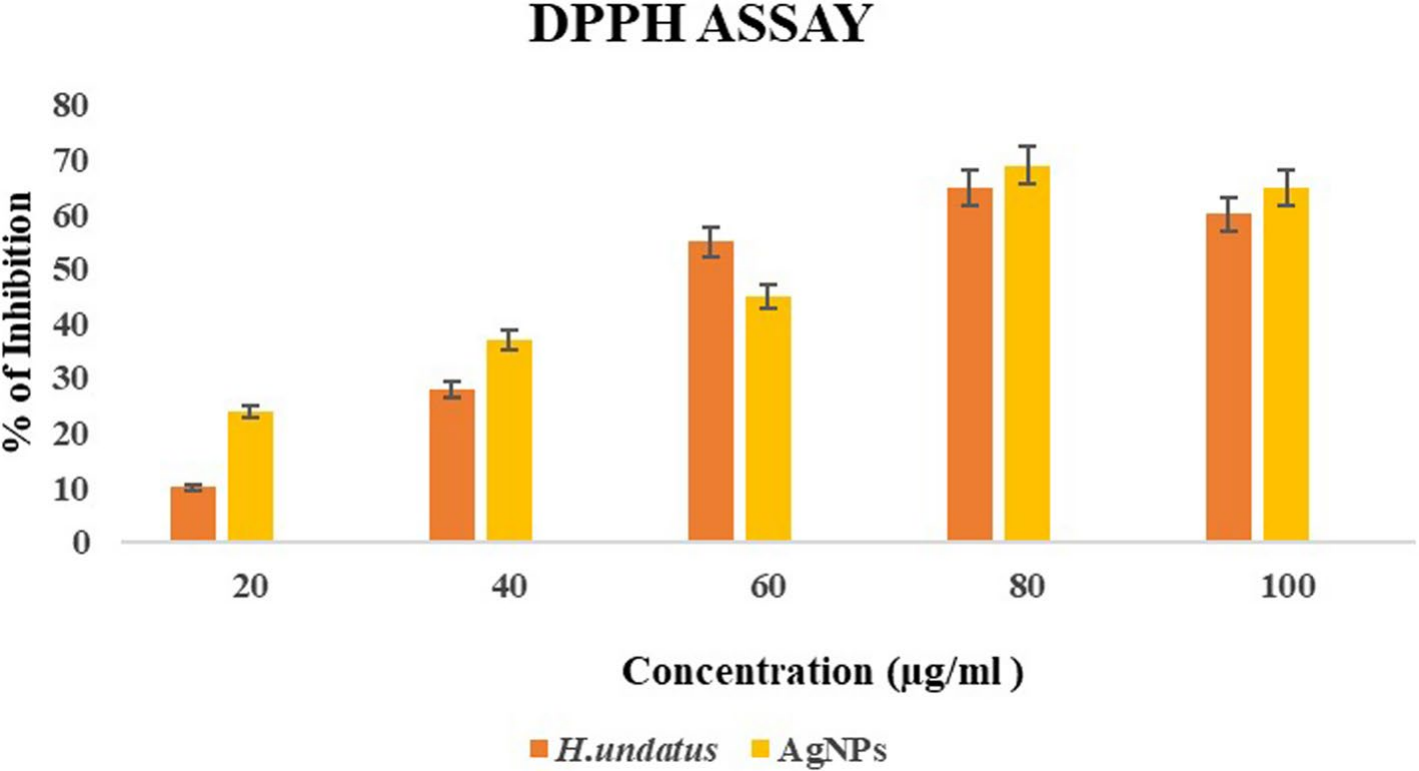
DPPH radical scavenging activity of *H. undatus* and AgNPs

**Fig. 13:**
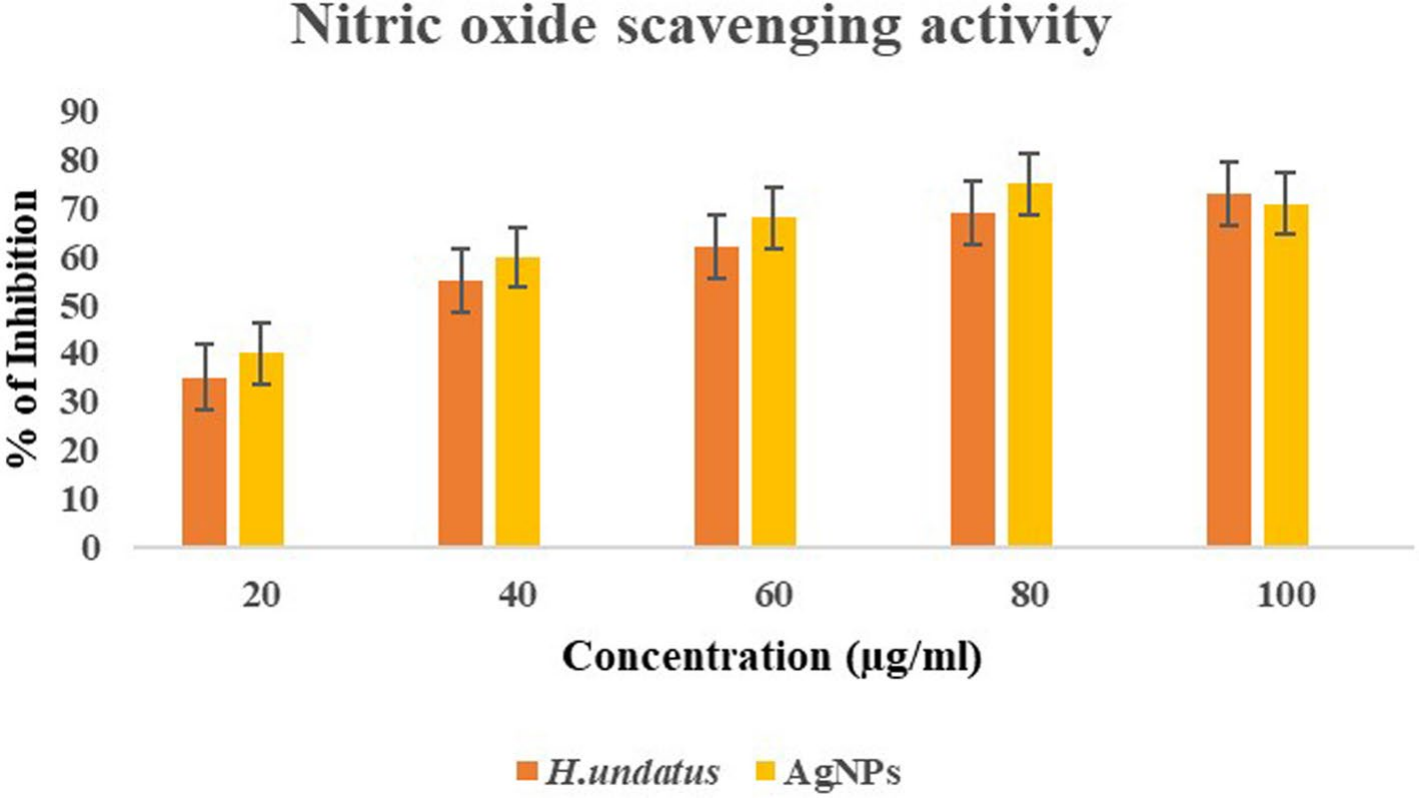
Nitric oxide radical scavenging activity of *H. undatus* and AgNPs

The antioxidant activity of the AgNPs and *H. undatus* peel extracts was determined by the DPPH method. The AgNPs and *H. undatus* peel extracts were compared based on their resulting IC_50_ values. AgNPs Silver nanoparticles exhibited maximum radical scavenging activity (IC_50_ of 3.8 µg/mL), while *H. undatus* peel extract exhibited the IC_50_ value of 2.03 µg/mL (Fig. 12). The inhibition % of nitric oxide radical scavenging activity by the AgNPs from *H. undatus* at different concentrations (20, 40, 60, 80, and 100 µg/mL) was compared based on the resulting IC_50_ values. AgNPs exhibited maximum radical scavenging activity (IC_50_ 2.8 µg/mL), while the *H. undatus* peel extract exhibited an IC_50_ value of 2.3 µg/mL (Fig. 13). There is a growing demand for the development of cost-effective, eco-friendly techniques for synthesizing metallic nanoparticles with high yields and low toxicity. Several studies have reported the reduction of silver ions into AgNPs using plant extracts. It contains biologically active phytochemicals such as terpenoids, flavonoids, vitamins, and phenolics which are known for their antioxidant properties [35]. These compounds have a diverse range of biological activities and help to protect cells from damage caused by reactive oxygen species. Our study provides clear evidence that the synthesized NPs and fruit peel extract exhibited radical scavenging activity. Compared with fruit peel extract, AgNPs showed promising antioxidant properties. The presence of numerous bioactive compounds in fruit extract may be the reason for its antioxidant activity [36]. The interaction of plant metabolites with metal ions can lead to the production of enhanced compounds that scavenge free radicals. Negatively charged phytochemicals and positively charged AgNPs work together to enhance the bioactivity of plants through electrostatic interactions [34]. Previous research has also indicated that antioxidant activity tends to increase as treatment doses increase [37].

### Anticancer activity

**Fig. 14:**
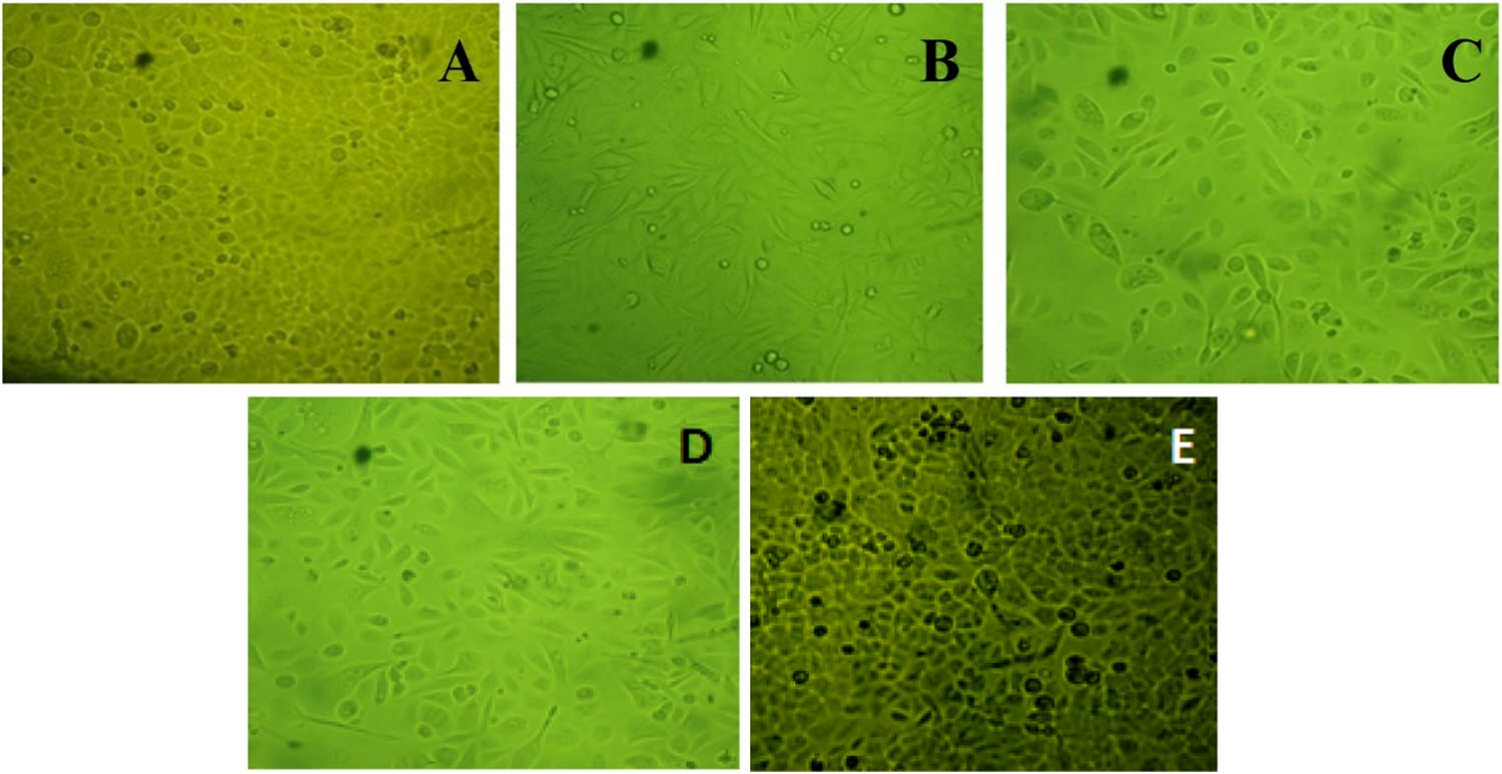
**A**–**C** Different concentrations (21, 25, and 55 µL) of *H. undatus* peel extract used to treat the PC3 cell line, **D** Positive control (Doxorubicin treated). **E** Negative control (Untreated)

**Fig. 15:**
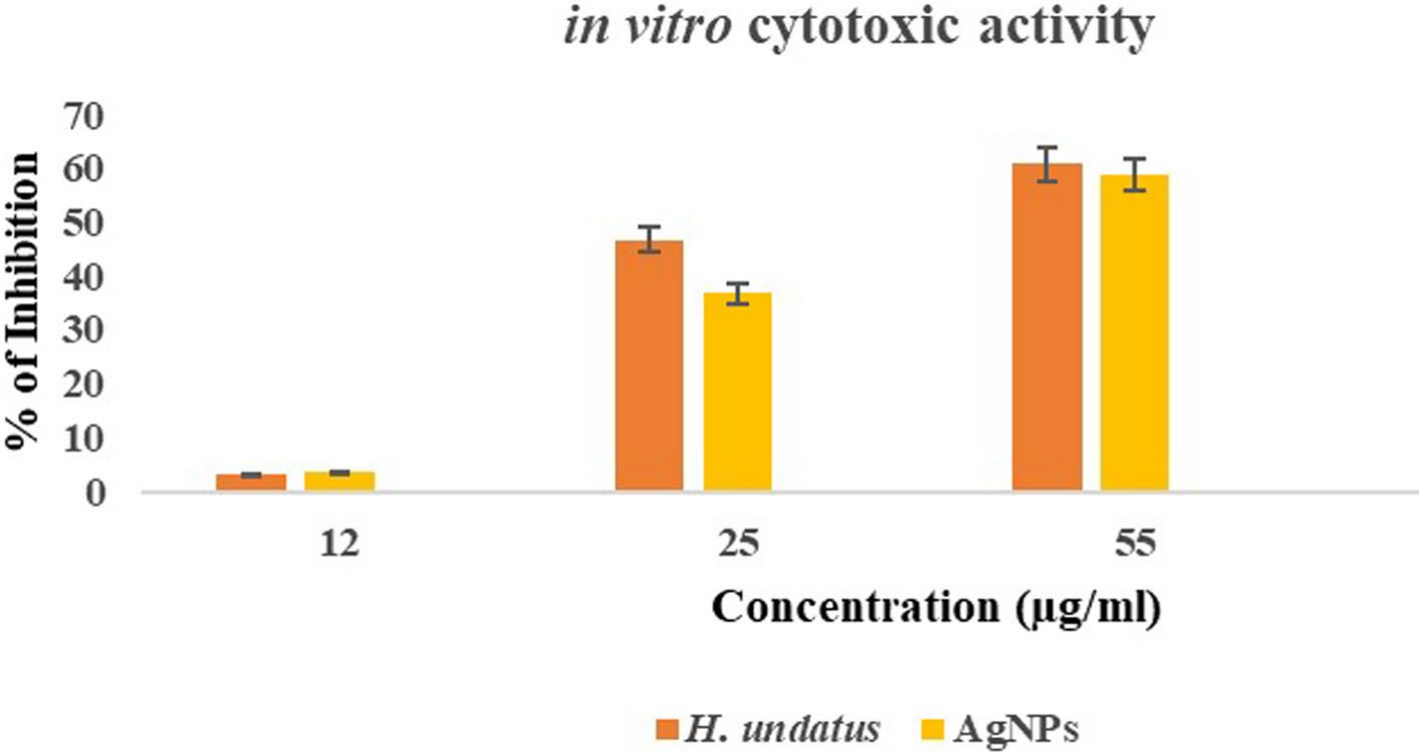
in vitro cytotoxic effects of AgNPs and *H. undatus* peel extracts on the PC3 cell line

The MTT assay was used to determine the cytotoxicity of the AgNPs and *H. undatus* peel extract on the PC3 cell line and to determine the viability of the cells. A significant degree of cytotoxicity was noted in cells treated with doxorubicin (positive control). However, a large number of cells was seen in the case of untreated cells (negative control) (Fig. 14). This assay was performed using three different concentrations (21, 25, and 55 µL) of AgNPs (Table 2) and *H. undatus* peel extract (Table 3). The IC_50_ results clearly showed that the tested AgNPs exhibited the minimum cytotoxic activity (IC_50_ of 0.2 µg/mL), while *H. undatus* peel extract exhibited the maximum cytotoxic activity (IC_50_ 0.3 µg/mL) (Fig. 15). The presence of bioactive compounds as capping agents in the green synthesis of AgNPs may account for the improved cytotoxic effects. Increasing the concentration of AgNPs and fruit peel extract leads to an increase in cytotoxicity. The anticancer property of AgNPs was due to the activation of reactive oxygen species. This activation leads to oxidative damage to cellular components such as DNA, proteins, and lipids, ultimately resulting in cell death [17]. The fruit extracts of *H. udantus* contain polyphenolic compounds that are adsorbed onto the surface of AgNPs. Along with the green synthesis of AgNPs, these biomolecules have been proposed as a potential solution for combating cancer cells in vitro due to their anticancer activity.

**Table 2:** in vitro cytotoxicity of the AgNPs against PC3 cell line

S. no.	AgNPs (µg/mL)	Absorbance of control at 570 nm	Absorbance of sample at 570 nm	% of inhibition
1	12	0.568	0.553	2.64
2	25	0.568	0.359	36.79
3	55	0.568	0.235	58.62

**Table 3:** in vitro cytotoxic effects of the *H. undatus* peel extract on the PC3 cell line

S. no.	*H. undatus* peel extract (µg/mL)	Absorbance of control at 570 nm	Absorbance of sample at 570 nm	% of inhibition
1	12	0.568	0.549	3.34
2	25	0.568	0.303	46.65
3	55	0.568	0.223	60.73

## Conclusion

Nanosized silver particles have attracted an increased amount of attention due to their physiochemical and optical properties. In the present study, AgNPs were successfully obtained from the reduction of silver nitrate by *H. undatus* peel extract. The fruit peel extract significantly contains the flavonoids and phenolics that are responsible for its antioxidant activity. The potential use of AgNPs obtained from *H. undatus* peel extract as an effective anticancer agent against PC3 cells has been suggested. The biosynthesized AgNPs from *H. undatus* peel extract showed significant antibacterial, antioxidant, anticancer, and thrombolytic activity which indicates their potential therapeutic applications. Therefore, further studies are required to discover medicinal drugs from this source.

## Data Availability

The authors declare that the data supporting the findings of this study are available within the paper and its supplementary information files.
